# Mold Size Effect in Microscale Laser Dynamic Flexible Bulging Assisted by Laser Pre-Shocking

**DOI:** 10.3390/mi13050757

**Published:** 2022-05-11

**Authors:** Yijun Fang, Pin Li, Xijin Zhen, Jindian Zhang, Zongbao Shen

**Affiliations:** 1 School of Agricultural Engineering, Jiangsu University, Zhenjiang 212013, China; fang_yijun@163.com; 2 School of Mechanical Engineering, Jiangsu University, Zhenjiang 212013, China; zjd965138386@163.com (J.Z.); szb@ujs.edu.cn (Z.S.); 3 Shipbuilding Technology Research Institute, Shanghai 200032, China; zxj20laser@163.com

**Keywords:** microscale laser dynamic flexible bulging, mold size effect, laser pre-shocking, necking, forming uniformity

## Abstract

The size effect seriously affects the forming quality of micro-formed parts in the field of micro-forming. This paper focuses on the influence of the mold size effect in microscale laser dynamic flexible bulging (μLDFB). The experimental results indicate that, for the copper foil with a given thickness, there are suitable mold characteristic sizes to obtain better forming quality. The surface quality of bulging parts is poor when the mold characteristic size is small. However, the forming symmetry and forming uniformity of bulging samples are reduced when the mold characteristic size is large. As the laser pulse energy increases, the plastic strain increases, and the bulging samples experience five stages: uniform plastic deformation, local necking, cracks in the bulging zone, complete fracture in the bulging zone and complete rupture at the mold entrance zone. The increase of the surface roughening rate caused by the increase of grain size and mold characteristic size makes local necking easier, which further leads to fracture. On this basis, in this paper laser pre-shocking (LPS) is introduced to improve the forming quality. Comparative experiments show that LPS has a positive effect on improving the surface quality and the forming performance of bulging samples. The forming limit of bulging samples is increased and the occurrence of local necking is delayed.

## 1. Introduction

Microscale laser dynamic forming is a new kind of micro-forming technology [1]. When the pulse laser irradiates the workpiece surface, the surface material is rapidly vaporized and ionized to produce the plasma. When the plasma expands and explodes, a strong shock wave is generated, which acts on the flexible medium to produce elastic deformation. The elastic medium pressurizes and transmits the shock wave to the workpiece according to the impedance mismatching effect [2]. When its pressure is higher than the yield strength of the workpiece, plastic deformation occurs. Microscale laser dynamic forming avoids the alignment problem in traditional micro-forming, and has the advantages of high forming efficiency, no pollution and high forming precision, which has become a research hotpot nowadays [3].

However, the existence of size effect in micro-forming restricts the development of micro-forming technology. The flow stress, plastic flow and other performances of materials of parts with miniature geometric size are very different from those of macro-scale parts. However, when the grain number in the workpiece thickness direction decreases to a certain value, the failure behaviors and mechanical properties of micro-formed parts are significantly affected by the workpiece thickness and grain size. Therefore, t/d (t is the workpiece thickness and d is grain size) is an important parameter used to study the size effect in micro-forming [4,5,6]. Miyazaki et al. [7] studied the effect of grain size and workpiece thickness of copper, aluminum, iron and other materials on bulging in tensile test. The test results show that the relationship between flow stress and grain size of copper, aluminum and other materials is related to the value of t/d. When the critical value of t/d is less than the that of the workpiece, the flow stress decreases with the decrease of workpiece thickness. Wang et al. [8] carried out micro tensile tests and micro channel flexible forming tests on pure nickel sheet with different grain sizes and workpiece thicknesses. It was found that when t/d < 1.2, the flow stress, strength and fracture strain of nickel plate are significantly reduced. When t/d decreases from 1.8 to 0.8, the fracture mode changes from dimple slip fracture to pure slip fracture. When t/d < 1.2, serious thickness thinning and surface coarsening occurred due to the strong local deformation. In particular, when there is only one single grain in the thickness direction, the specific grain orientation will lead to serious local deformation. Yuan et al. [9] studied the mechanical properties of aluminum plates with different thickness by combining the methods of experimental and numerical simulation. The results show that after uniaxial tensile test, when the thickness decreases to a certain value, the fracture mode of aluminum plate changes from brittle fracture to ductile fracture, and the fracture strain and tensile strength decrease with the decrease of aluminum plate thickness, which can be attributed to the number of activated slip systems being related to the fracture strain.

The mold size effect is also significant. Ghassemali et al. [10] investigated the effect of mold size on progressive micro-forming processing of copper foil. The results show that the microparts with large and small mold characteristic sizes have forming defects, and there are appropriate mold characteristic sizes to obtain better forming quality. Kim et al. [11] studied the size effect of copper foil in micro imprinting, focusing on the influence of the ratio of mold characteristic size to grain size. Zheng et al. [12] studied the progressive forming process of conical flange parts of metal foil under the experimental conditions of different mold characteristic size and grain size. It was found that the conical surface of coarse-grained samples is poor, and the geometric defects and surface quality deteriorate significantly as the mold characteristic size reduces.

At present, the research on size effect is mostly focused on static/quasi-static loading conditions, and mostly through uniaxial tensile experiments. There are still few studies on size effect under high strain rate deformation. Escobedo et al. [13] studied the effects of grain size and grain boundary characteristics on the dynamic tensile test of copper with different thickness, and proposed a new void growth and coalescence mechanism. The experiment results found that the resistance of grain boundaries with different orientations to void formation is different, and the voids grow preferentially at the grain boundaries with incorrect high angle orientation. Zhu et al. [14] conducted tensile tests of pure titanium foil with different grain sizes under different strain rates. It was found that the flow stress decreases with the decrease of t/d, and the fracture mode showed great differences with the change of strain rate. Under the condition of low strain rate, ductile fracture and cleavage fracture existed at the same time. When the strain rate increased to 1500 s^−1^, it was a typical ductile fracture mode. At the same time, the size effect seriously affects the fracture behavior of the workpiece. The fracture strain is positively proportional to the grain size. The reduction of t/d ratio and the increase of surface roughness will aggravate the uneven deformation and eventually lead to fracture [15]. Gu et al. [16] studied the size effect in laser shock hydraulic micro-forming and found that when t/d > 1, with the increase of grain size and the decrease of workpiece thickness, the overall thickness thinning rate increases and local necking occurs in the bulging transition zone. When t/d < 1, the thickness distribution of micro bulging parts is poor and the surface quality deteriorates sharply. The influencing factors of size effect under high strain rate micro-forming in this paper are more complex, thus it is necessary to consider the coarsening of the free surface of the workpiece in the process of micro-forming. Surface coarsening has a great impact on micro-forming technology and product quality in micro plastic deformation. Yoshida et al. [17] simulated the tensile test of polycrystalline sheet through crystal plasticity model, and studied the effect of surface roughness on local necking. It was found that the free surface roughening is mainly caused by the difference of adjacent grain orientation. When the grain size becomes larger, the local strain concentration and necking occur easier. Cheng et al. [18] found that the PMC model without considering the surface roughness and fracture mode transition in metal micro-forming is no longer suitable for predicting the microscale forming limit, and the improved model is in better agreement with the experimental effect. Bong et al. [19] proposed a grain plastic finite element analysis method considering surface roughening, which is more consistent with the experimental results of forming limit diagram of uniaxial tensile test of ultra-thin ferritic stainless steel plate with thickness of only 0.1 mm, while the finite element analysis method without considering surface roughening is completely different.

Laser shock is one of the effective methods to suppress the defects of micro-formed parts caused by size effect and surface roughening. This is because laser shock plays a positive role in improving the surface roughness and refining the internal grains of the workpiece [20,21]. In our previous work, Zhang et al. investigated the grain size effect in micro-punching [22] and micro-bulging processes [23], and found that laser pre-shocking (LPS) effectively improved the forming quality of micro-parts. However, the existence of the mold size effect restricts the development of microscale laser dynamic flexible bulging (μLDFB). In this paper, the mold size effect in μLDFB is investigated, and LPS is introduced to reduce the mold size effect in μLDFB. The influence of LPS on the forming quality of micro-bulged parts is studied from the aspects of forming depth, surface roughness, forming uniformity, microstructure and nano-hardness.

## 2. Experiment

**Figure 1:**
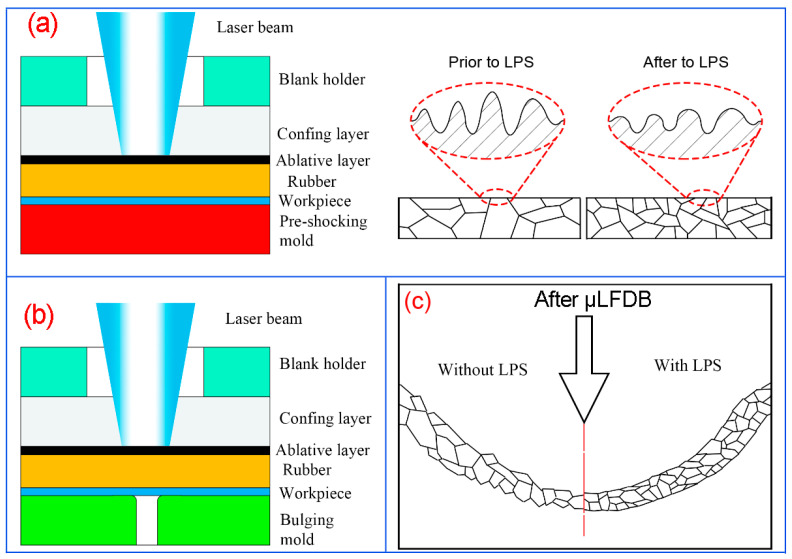
Schematic of microscale laser dynamic flexible bulging (μLDFB). (**a**) LPS; (**b**) μLDFB; (**c**) effect of LPS on the bulging samples.

The schematic of LPS process is shown in Figure 1a, which includes a blank holder, confining layer, ablative layer, rubber, workpiece and pre-shocking mold. Additionally, the schematic of the μLDFB process is shown in Figure 1b. In order to investigate the influence of mold characteristic size on the forming quality of bulging samples, bulging molds with three different diameters through-hole structures were used. To prove the feasibility of reducing the mold size effect using LPS, the comparative experiments were carried out, as shown in Figure 1c.

### 2.1. Experimental Materials

**Figure 2:**
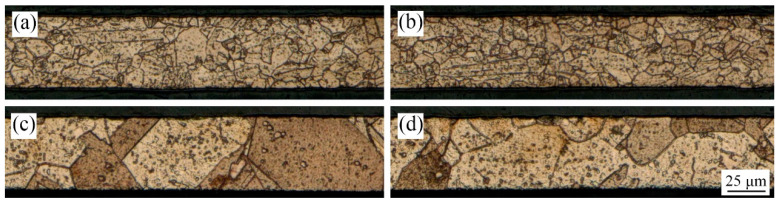
Microstructures of copper foils. (**a**) 450 °C annealed, without LPS; (**b**) 4500 °C annealed, with LPS; (**c**) 650 °C annealed, without LPS; (**d**) 650 °C annealed, with LPS.

The T2 copper foils with thickness of 50 μm annealed at 450 °C and 650 °C were used as the experimental material in this paper. The copper foil was evenly cut into 10 mm × 10 mm, and was cleaned with alcohol before the experiment. In the LPS experiment, copper foils were shocked by laser pulse energy of 447 mJ, as shown in Figure 1a. The metallographic corrosion of copper foil before and after LPS was carried out, and the obtained metallographic structures are shown in Figure 2. The results of grain size calculated by the cut-off point method are shown in Table 1. Grain refinement can be obviously observed after LPS.

### 2.2. Bulging Mold

**Figure 3:**
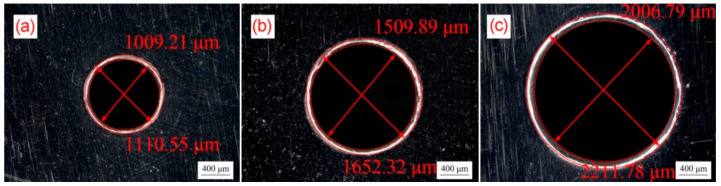
Real product photo of bulging molds. (**a**) λ = 0.25; (**b**) λ = 0.5; (**c**) λ = 1.

The design sizes of bulging molds are shown in Table 2, and the actual sizes of the prepared molds are shown in Figure 3. The experimental conditions and parameters of μLDFB demanded that the mold must have sufficient rigidity and hardness to stand strong shock load. AISI 1090 high carbon steel is selected as the mold material, and its hardness can reach 65 HRC after heat treatment, which meets the requirements of the experimental process. Meanwhile, the featureless smooth surface of high carbon steel is used as the pre-shocking mold.

### 2.3. Experimental Parameters and Equipment

**Figure 4:**
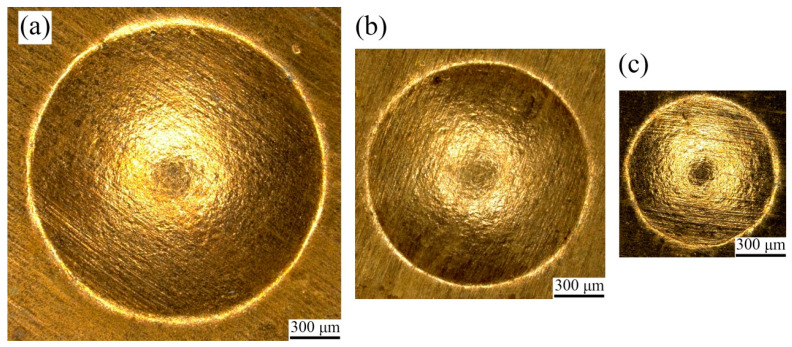
Topography of fine-grained bulging samples under laser pulse energy of 363 mJ. (**a**) λ = 1; (**b**) λ = 0.5; (**c**) λ = 0.25.

**Figure 5:**
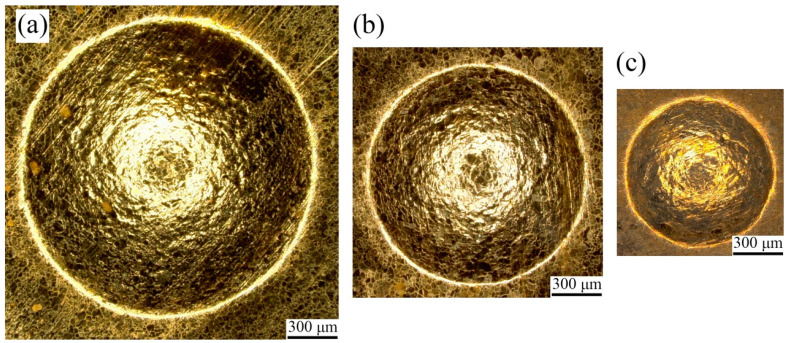
Topography of coarse-grained bulging samples under laser pulse energy of 363 mJ. (**a**) λ = 1; (**b**) λ = 0.5; (**c**) λ = 0.25.

The laser selected in the experiment is the Spitlight 2000 Nd: YAG nanosecond laser, and its technical parameters are shown in Table 3. Specifically, black paint was selected as the ablative layer, the confining layer was polymethyl methacrylate (PMMA), and polyurethane soft film was selected as the flexible medium. Related experimental parameters are shown in Table 4. The surface topographies of the fine-grained and coarse-grained bulging samples obtained by different bulging molds are shown in Figure 4 and Figure 5, respectively, and the bulging samples are well formed.

After the experiment, cold mounting was carried out to obtain the section morphology of bulging samples, and the mixed solution of FeCl_3_, HCl and deionized water with the configuration ratio of 5:15:85 was used for metallographic corrosion to obtain the microstructure morphology. Digital microscopy (KEYENCE VHX-1000C) and SEM (Hitachi S-3400) were used to observe the surface morphology of bulging samples, laser confocal microscope (KEYENCE VK-X200) was used to measure the surface roughness and Agilent Nano Indenter G200 nano indentation in situ nanomechanical testing system was used to measure the nano-hardness and elastic modulus.

## 3. Results and Discussion

### 3.1. Forming Depth

**Figure 6:**
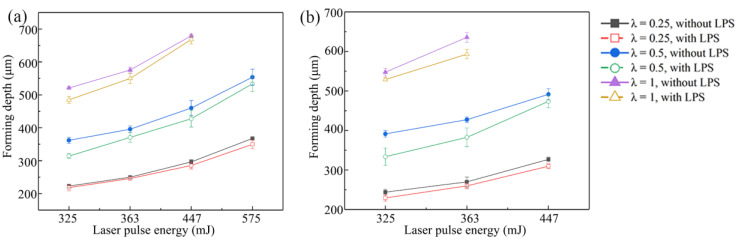
Effect of mold characteristic size on the forming depth of bulging samples. (**a**) Annealed at 450 °C; (**b**) annealed at 650 °C.

The maximum depth of the bulging area is defined as forming depth in this section, and five bulging samples were measured under each experimental condition to obtain the average forming depth. Figure 6 shows the influence of mold characteristic size on forming depth of bulging samples under different grain sizes and laser pulse energies. It can be found that the forming depth increases with the increase of laser energy. Specifically, when the laser energy increases from 325 to 575 mJ, the forming depth of bulging samples annealed at 450 °C without LPS increases from 223.2 to 367.7 μm. In addition, the variation trends of forming depth of both coarse-grained and fine-grained samples are similar under the same mold characteristic size. The forming depth of bulging samples increases with the increase of scale factor λ. Specifically, when the laser energy is 363 mJ, the forming depths of fine-grained bulging samples without LPS fabricated by three bulging molds are 250.0 μm, 395.6 μm and 575.5 μm, respectively. Large mold characteristic size is conducive to enhancing the micro-forming ability of the workpiece [24]. The forming depth of bulging samples is small because the plastic deformation of copper foil is dominated by slip when λ = 0.25. The reduction of mold characteristic size means that the number of grains and grain boundaries in the bulging zone are reduced, which leads to the reduction of flow stress [25]. The deformation coordination ability between grains is reduced, resulting in the reduction of slip. At the same time, the constraint of mold cavity also leads to the reduction of plastic strain. In the micro-bulging experiment of the same mold, the forming depth increases with the increase of the grain size. This is because the yield strength and deformation resistance of the workpiece decrease with the increase of grain size, and the plastic deformation is more intense [26]. In the micro-bulging experiments of all molds, the forming depth of bulging samples with LPS is reduced. This can be attributed to the fact that LPS reduces the flow stress in the sample by refining the grain, improves the yield strength, and improves the hardness and fatigue resistance of the sample [27,28]. It is worth noting that, when λ = 0.25, the reduction of forming depth of bulging samples is very limited, which is due to the small plastic deformation requiring less grain rotation and slip, and the effect of grain refinement is not obvious.

### 3.2. Surface Roughness

**Figure 7:**
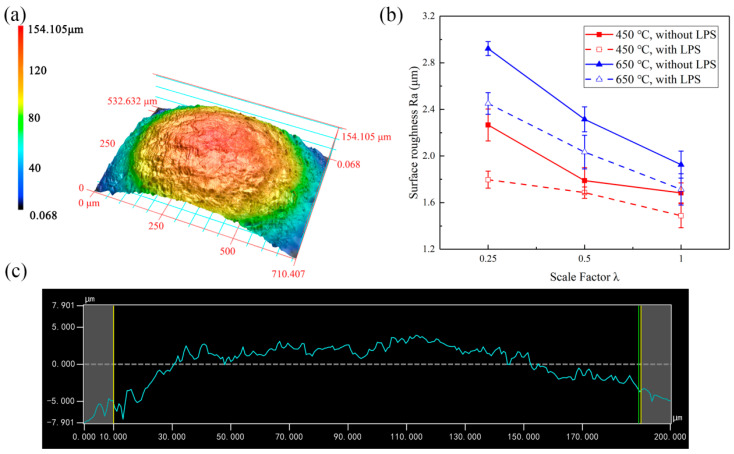
(**a**) 3D topography of bulging samples; (**b**) measurement value of surface roughness of bulging samples; (**c**) roughness curve.

The surface 3D topographies and roughness curves observed by KEYENCE VK-X200 laser confocal microscope are shown in Figure 7a,c. The measured surface roughness (Ra) is the roughness at the bulging center zone bottom of the bulging sample. The influence of grain size and mold characteristic size on surface roughness is shown in Figure 7b. It can be seen that the surface roughness decreases with the increase of mold characteristic size when the laser energy is 363 mJ. Specifically, the surface roughness of the bulging sample without LPS annealed at 450 °C decreases from 2.267 to 1.684 μm with an increase of 25.7%, and surface roughness of the bulging sample without LPS annealed at 650 °C decreases from 2.922 to 1.926 μm with an increase of 34.1%. With the decrease of mold characteristic size, the number of grains involved in micro-bulging decreases, bulging surface roughening becomes more serious due to the grain rotation and relative slip caused by the misalignment of orientation between adjacent grains and the surface roughness increases [29]. At the same time, the surface roughness of coarse-grained samples is generally high, which is consistent with the morphology comparison between Figure 4 and Figure 5. This can be attributed to the fact that the surface roughening rate increases with grain size and plastic strain under the same mold deformation conditions [30,31].

The surface quality of coarse-grained samples is poor due to the high surface roughening rate. However, the surface roughness of bulging samples with LPS generally decreases and the surface quality is improved. Specifically, when λ = 0.25, the surface roughness of the bulging samples annealed at 450 °C decreases from 2.267 to 1.797 μm with an increase of 20.7%, and the surface roughness of the bulging samples annealed at 650 °C decreases from 2.922 to 2.451 μm with an increase of 16.1%.

### 3.3. Microstructure Morphology

**Figure 8:**
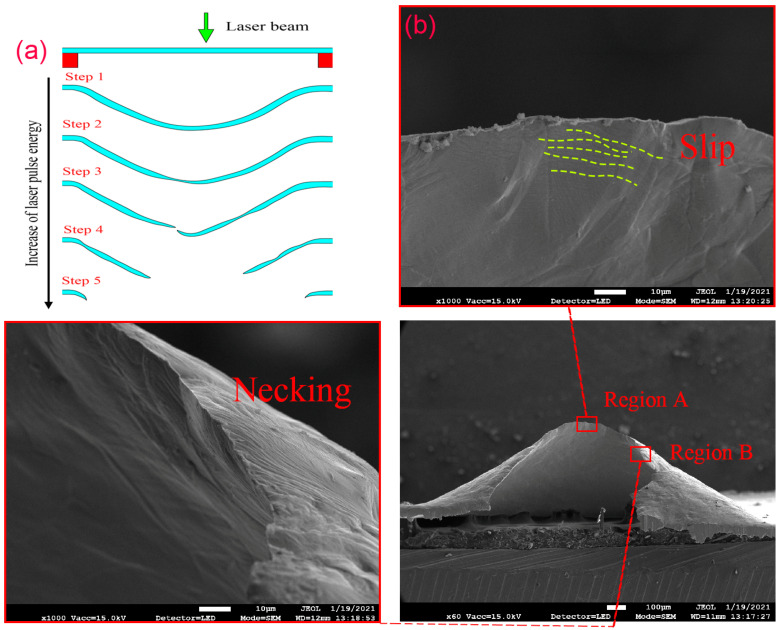
(**a**) Schematic diagram of μLFDB and fracture mode under different laser pulse energies; (**b**) SEM morphology of fractured parts annealing at 650 °C.

**Figure 9:**
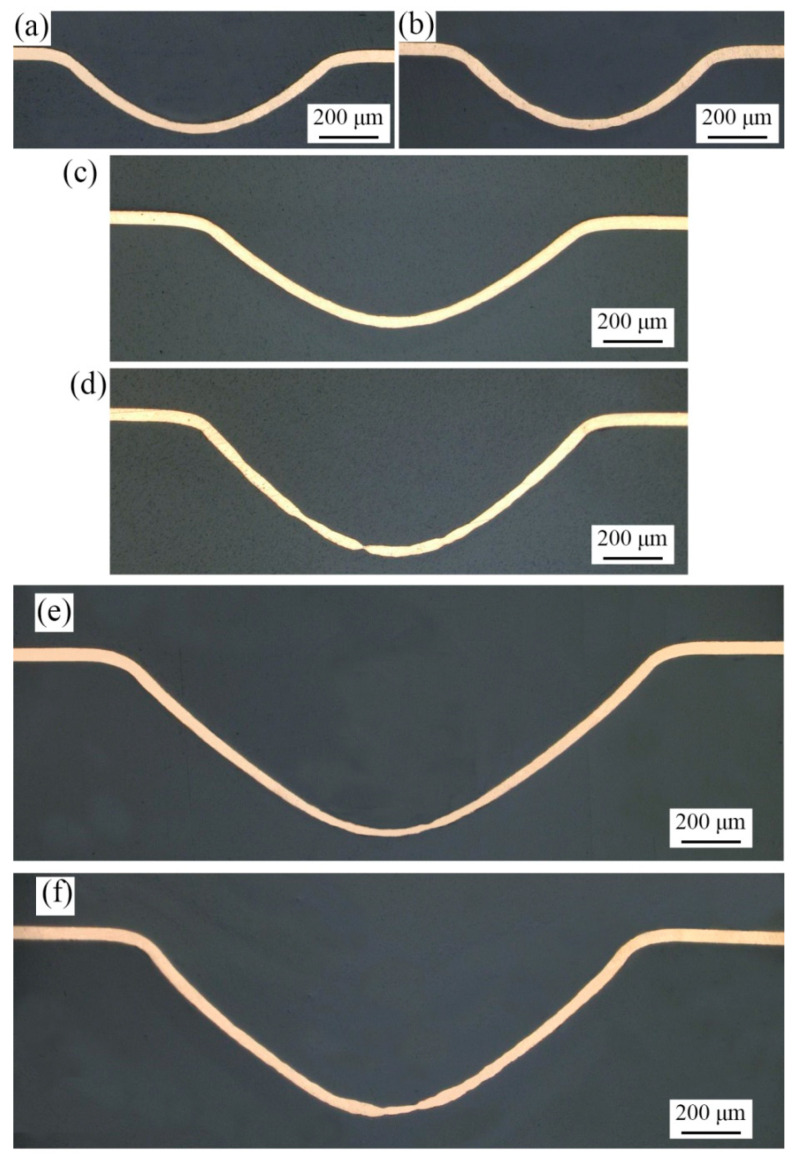
Section morphologies of bulging samples under laser pulse energy of 447 mJ. (**a**) λ = 0.25, fine-grained samples; (**b**) λ = 0.25, coarse-grained samples; (**c**) λ = 0.5, fine-grained samples; (**d**) λ = 0.5, coarse-grained samples; (**e**) λ = 1, fine-grained samples; (**f**) λ = 1, coarse-grained samples.

The section morphologies of bulging samples under different laser shock energies are observed. The influence of laser pulse energy on bulging samples is shown in Figure 8a. The plastic strain increases with the increase of laser energy, and the deformation process of bulging sample can be summarized as five stages: uniform plastic deformation, local necking, cracks in the bulging zone, complete fracture in the bulging zone and complete rupture at the mold entrance zone. The grain size and mold characteristic size seriously affect the formability of bulging samples. As shown in Figure 9, when the laser pulse energy is 447 mJ, the fine-grained bulging sample is in stage 1, while the coarse-grained bulging sample is already in stage 2 when λ = 0.5. Meanwhile, the fine-grained bulging sample is in stage 1 when λ = 0.25 and 0.5, and it is already in stage 2 when λ = 1. Therefore, too large a grain size and mold characteristic size are not conducive to the micro-bulging technology of metal foil in the micro-forming process. Combined with the previous section, too small a mold characteristic size leads to poor surface quality of bulging sample. Therefore, for metal foil with specific grain size and workpiece thickness, there is an appropriate mold characteristic size to obtain better surface quality and formability.

**Figure 10:**
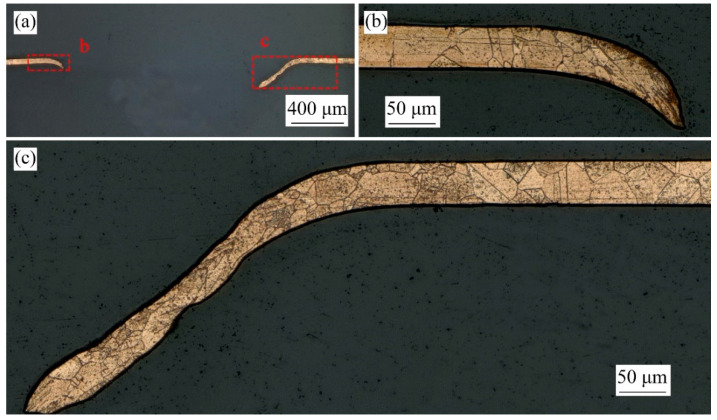
(**a**) λ = 1, microstructure of the bulging sample without LPS of mold 3 annealed at 650 °C under laser pulse energy of 1050 mJ; (**b**,**c**) correspond to region b and region c in (**a**).

As shown in Figure 8b, through the observation of the section morphology of the fractured bulging sample in stage 3, it is found that there is serious thickness thinning (region B) before the fracture, and a lot of slip traces (region A) are found at the crack tip. Therefore, the fracture of the bulging sample belongs to typical ductile fracture, and local necking is the precursor of the crack [32]. The microstructure morphology of the fracture bulging sample without LPS in stage 4 is shown in Figure 10. It can be found that under the combined action of laser shock wave pressure, blank holder force and rigid constraint of mold, obvious stress concentration occurs at the entrance of the mold (Figure 10b), and obvious grain refinement occurs on the upper and lower surfaces of the sample. In the bulging zone, local necking often occurs at the position with uneven grain size distribution and large difference in grain orientation. The difference is that grain refinement only occurs on the upper surface of the workpiece, because the laser shock wave attenuates along the thickness direction when acting on the workpiece [33]. The closer the bulging center, the greater the plastic strain, and the easier it is for local necking to develop into cracks. When the grain size decreases, the flow stress of the workpiece increases, the coordination ability between grains also increases, the fracture strain of the workpiece increases, and the local necking is alleviated [34]. Therefore, grain size refinement and grain size distribution homogenization are effective means to improve the formability of workpiece.

**Figure 11:**
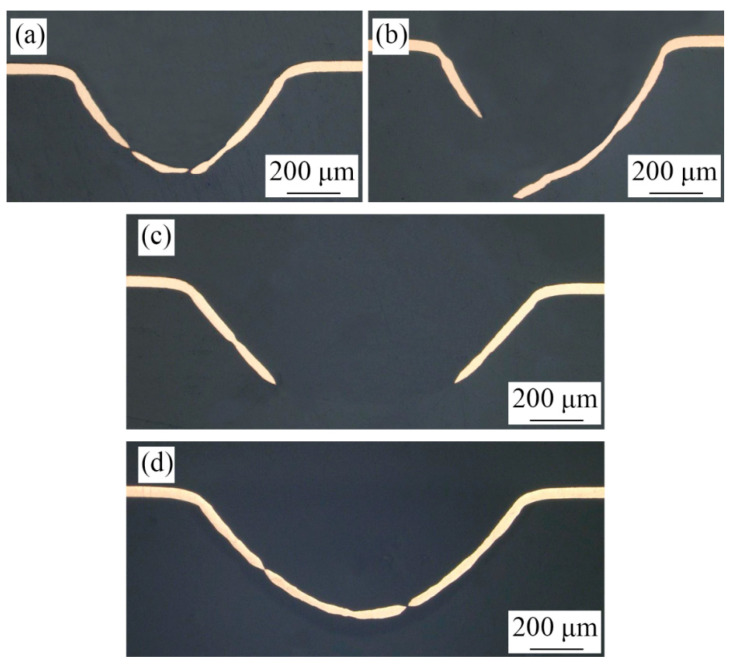
Effect of LPS on the section morphologies of bulging samples annealed at 650 °C. (**a**) λ = 0.25, 725 mJ, without LPS; (**b**) λ = 0.25, 725 mJ, with LPS; (**c**) λ = 0.5, 575 mJ, without LPS; (**d**) λ = 0.5, 575 mJ, with LPS.

As shown in Figure 11a,b, when the laser pulse energy is 725 mJ, the bulging sample without LPS fabricated by mold 1 is in stage 4 and the bulging sample with LPS is in stage 3. Similarly, as shown in Figure 11c,d, when the laser pulse energy is 575 mJ, the bulging sample without LPS fabricated by mold 2 is in stage 4 and the bulging sample with LPS is in stage 3. Therefore, LPS can be used to alleviate the local necking and other phenomena caused by surface roughening due to its positive role in improving the formability of workpiece, and further studies of the effect of LPS on the forming quality of bulging samples is needed.

### 3.4. Forming Symmetry

**Figure 12:**
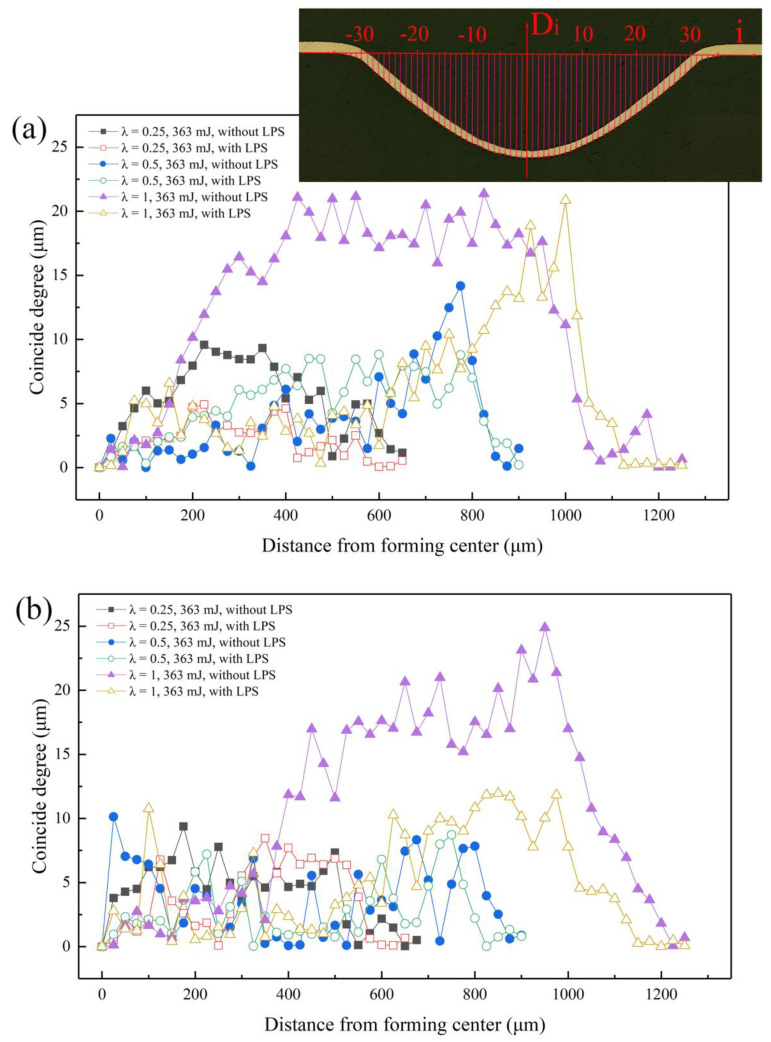
Effect of mold characteristic size on the coincidence degree C of bulging samples under laser pulse energy of 363 mJ. (**a**) 450 °C annealed; (**b**) 650 °C annealed.

In order to investigate the influence of LPS and other parameters on the forming symmetry of bulging samples, the forming profile distribution of bulging samples is quantitatively analyzed by measuring the forming depth at each position of the bulging zone. Every 25 μm was measured three times in the forming depth and the average value was obtained. Figure 12 shows the measurement method. The coincidence degree C is defined to quantify the forming symmetry of bulging samples, which defined as:

(1) $$C=\left|D_{i}-D_{-i}\right|$$

where $D_{i}$ represents the forming depth measured at the selected point. Taking the forming depth at the bulging center as $D_{0}$, $D_{i}$ and $D_{-i}$ are bulging zones symmetrical around the bulging center. Therefore, the coincidence degree C is selected as the criterion. It can be seen that the forming symmetry of bulging samples become better with the decrease of coincidence degree C.

According to the measured data, Figure 12 shows the effect of mold characteristic size on the forming symmetry of bulging samples under the same laser energy. When λ = 0.25, the maximum coincidence degree C of both coarse-grained and fine-grained samples appears in the bulging transition zone, and its values are 9.58 μm and 9.37 μm, respectively, which is equivalent to the maximum coincidence degree C in the bulging zone when λ = 0.5, and its values are 8.86 μm and 10.13 μm. When λ = 1, the maximum coincidence degree C in the bulging zone rises sharply to 21.14 μm and 20.13 μm, respectively. Thus, the maximum coincidence degree C appears in the mold entrance zone when λ = 1, and its values are 21.36 μm and 24.88 μm, respectively. However, when λ = 0.5, the maximum coincidence degree C may appear in the mold entrance zone or in the bulging zone. The maximum coincidence degree C of fine-grained bulging samples in Figure 12a is 14.17 μm in the mold entrance zone. With the sharp increase of plastic strain, the bending deformation required to match the plastic deformation in the mold entrance zone needs to be more intense. At this time, the required grain rotation and slip are more intense, and the forming symmetry decreases significantly.

The improvement of forming symmetry by LPS becomes more and more obvious with the increase of mold characteristic size. When λ = 0.25, for fine-grained samples, the forming symmetry is improved, and the maximum coincidence degree C is reduced to 4.90 μm, but for coarse-grained samples, the reduction of the maximum coincidence degree C is not obvious, only reducing to 8.46 μm. When λ = 1, the coincidence degree in the bulging zone is greatly reduced to 13.73 μm and 11.96 μm, respectively. The decline reached 35.7% and 51.9%. This can be attributed to the fact that LPS can improve the uniformity of grain size distribution by refining the grain, inhibiting the surface roughening in plastic deformation to a certain extent and reducing the possibility of local stress–strain concentration in plastic deformation. Therefore, LPS plays a positive role in improving the forming symmetry of bulging samples.

### 3.5. Forming Uniformity

The increase and the uneven distribution of grain size lead to the sharp increase of surface roughening rate in the process of plastic deformation, resulting in local stress-strain concentration, which further leads to local necking. Therefore, it is necessary to study the thickness change of bulging samples. In this section, the thickness was measured and the thickness thinning rate of bulging samples was calculated to study the influence of LPS on the forming uniformity.

The KEYENCE VHX-1000C was used for three-dimensional measurement, the thickness in bulging zone was measured every 25 μm three times to obtain the average value. The thickness thinning rate T is defined as follows:

(2) $$T=\frac{l_{0}-l_{i}}{l_{0}}\times 100\%$$

where $l_{0}$ represents the initial thickness of the workpiece and $l_{i}$ is the thickness at each measurement spot of the bulging zone after μLDFB. The forming quality of bulging samples becomes better with the decrease of the thinning rate T.

**Figure 13:**
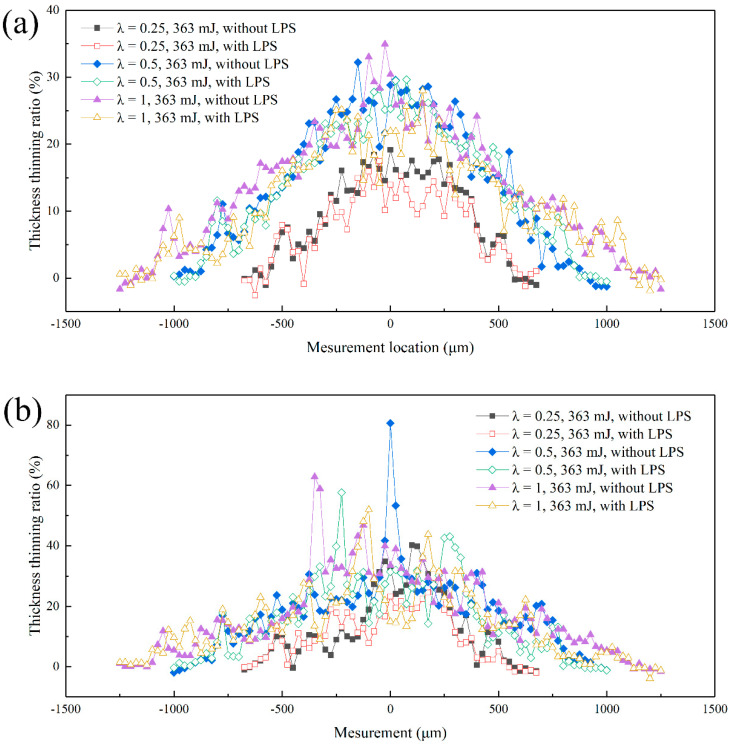
Thickness thinning rate of bulging samples with different molds under laser pulse energy of 363 mJ. (**a**) Fine-grained samples (450 °C annealed); (**b**) coarse-grained samples (650 °C annealed).

Figure 13 shows the effect of mold characteristic size on the thickness thinning rate of bulging samples under laser pulse energy of 363 mJ. For fine-grained samples (as shown in Figure 13a), the overall change trend is similar, and it increases generally from the mold entrance zone to the bulging center zone. The thickness thinning rate generally increases with the increase of the mold characteristic size, and the maximum thickness thinning occurs in the bulging center zone, reaching 19.2%, 32.2% and 34.9%, respectively, which is similar to the results of Liu et al. [35]. It can be found that the thickness thinning rate of bulging samples has little difference when λ = 0.5 and λ = 1. However, the change trend of coarse-grained samples tends to be abnormal (as shown in Figure 13b). The sharp growth point can be seen in the bulging transition zone, and the overall maximum thickness thinning rate increases significantly, reaching 40.2%, 80.6% and 62.8%, respectively. From the variation curve of thickness thinning rate, the bulging samples have obvious local necking in the bulging zone. This is because the bulging process is affected by the grain orientation and its size distribution when the grain size is close to the thickness of the workpiece. At this time, local stress concentration and uneven deformation occur easily, resulting in local necking and sharp thickness thinning.

**Figure 14:**
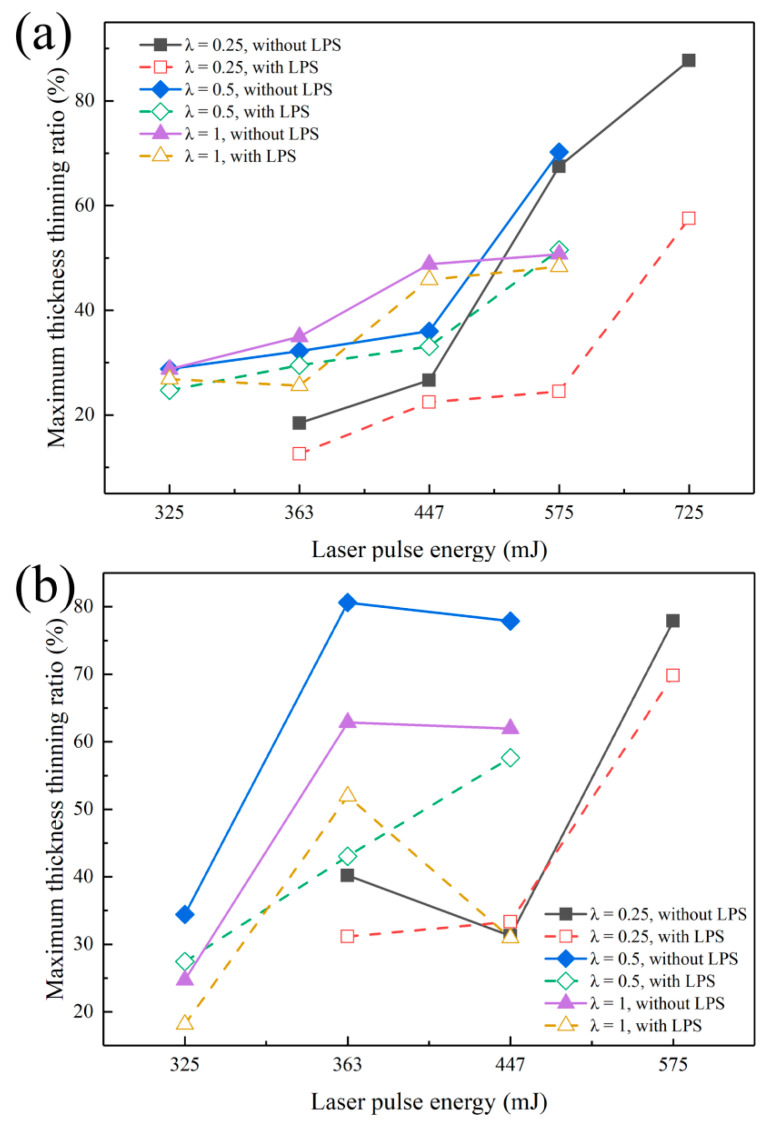
Maximum thickness thinning rate of bulging samples with different molds. (**a**) Fine-grained samples (450 °C annealed); (**b**) coarse-grained samples (650 °C annealed).

In this section, the maximum thickness thinning rate under different laser pulse energy is proposed to further explore the influence of mold characteristic size on the thickness thinning of bulging samples, and the specific values obtained are shown in Figure 14. It is found that the maximum thickness thinning rate of bulging samples is 87.7% (fine-grained samples, λ = 0.25, laser pulse energy of 725 mJ) and 80.6% (coarse grained samples, λ = 0.5, laser pulse energy of 363 mJ). Fracture has still not occurred when the maximum thinning rate is close to 80%, showing high formability. The superplastic deformation behavior of the bulging sample is mainly due to the fact that red copper is a high strain rate sensitive material, serious grain boundary slip and rotation occur in the bulging process [36] and the flow stress of the workpiece increases with the increase of strain rate [37]; the workpiece under the condition of high strain rate forming shows better forming ability. The strain rate of μLDFB is as high as 10^6^–10^7^ s^−1^. Therefore, the necking propagation is effectively delayed in the bulging process, and then the maximum thickness thinning rate is improved.

As shown in Figure 14a, for fine-grained samples, when λ = 0.25 and 0.5, the maximum thinning rate of bulging samples increases sharply with the laser pulse energy ranging from 447 to 575 mJ, which increases from 26.6% and 36.0% to 67.5% and 70.2%, respectively. This means the local necking occurs in bulging samples. The maximum thinning rate of bulging samples increases obviously when λ = 0.5, but it is still growing steadily when λ = 1. Therefore, the increase of mold aperture is conducive to delaying the occurrence of local necking. As shown in Figure 14b, for coarse-grained samples, the maximum thinning rate of bulging samples when λ = 1 is generally lower than when λ = 0.5. Although the plastic strain increases, the increase of the grain numbers in the bulging zone enhances the coordination ability of the workpiece in the process of plastic deformation. Therefore, the large mold characteristic size can promote the uniform distribution of strain on different grains, and local necking does not occur easily [29]. At the same time, it is noted that the fracture of bulging sample still does not occur under higher laser pulse energy when λ = 0.25. This can be attributed to the reduction of the grain number involved in plastic deformation meaning an increase of geometric dislocation density in the grains, which leads to local hardening of the bulging samples [38]. At this time, the deformation resistance increases significantly and the plastic strain decreases greatly (as shown in Figure 6). LPS generally reduces the maximum thinning rate of bulging samples and effectively suppresses the local necking phenomenon in the bulging process. When λ = 0.25 and 0.5, the maximum thinning rate of bulging samples increases sharply with the laser pulse energy increase from 447 to 575 mJ. Specifically, the maximum thinning rate of fine-grained bulging samples decreases from 67.5% to 24.5% (λ = 0.25, 575 mJ), and the maximum thinning rate of coarse-grained bulging samples decreases from 77.9% to 57.6% (λ = 0.5, 447 mJ).

**Figure 15:**
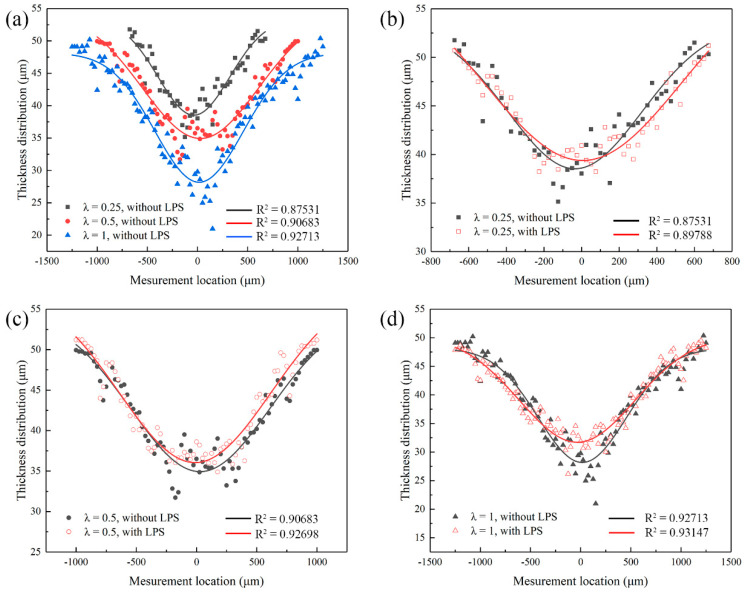
Effects of LPS and mold characteristic size on the thickness distribution of bulging samples annealed at 450 °C under laser pulse energy of 447 mJ. (**a**) Samples without LPS, λ = 0.25, 0.5, 1; (**b**) samples with and without LPS, λ = 0.25; (**c**) samples with and without LPS, λ = 0.5; (**d**) samples with and without LPS, λ = 1.

In order to further quantify the influence of mold characteristic size and LPS on the forming uniformity of bulging samples in the process of plastic deformation, the measured section thickness was regressed. It can be found from the regression equation that with the increase of the mold characteristic size, the deterministic parameter R^2^ of the regression equation increases (as shown in Figure 15a–d), and the thickness distribution is more uniform, but the minimum thickness decreases, which means that the bulging sample is more prone to local necking, resulting in fracture failure when λ = 1. It can be seen from regression analysis that the increase of the mold characteristic size is conducive to the improvement of the forming uniformity of bulging samples, but its improvement range is limited. Specifically, the thickness distribution of bulging samples has little difference when λ = 0.5 (as shown in Figure 15c) and 1 (as shown in Figure 15d). LPS has a positive effect on the thickness distribution of bulging samples. Similarly, the improvement of thickness distribution of bulging samples is limited when λ = 1, because the grain deformation in the large bulging zone is relatively uniform. However, the deterministic parameter R^2^ of the equation increases obviously with the decrease of the mold characteristic size. At this time, LPS is applied to the bulging process of small characteristic micro-parts, which can significantly improve the forming quality.

### 3.6. Nanoindentation Test

Hardness is the ability of the workpiece to resist local hard objects pressing into its surface. The degree of local plastic deformation on the material surface can be used to compare the softness and hardness of the tested material. The harder the material, the smaller the plastic deformation. Indentation hardness is widely used in engineering technology. Due to the fact that the thickness of the red copper foil used in this paper is only 50 μm, nanoindentation test was selected to obtain the nano-hardness and elastic modulus of bulging samples.

**Figure 16:**
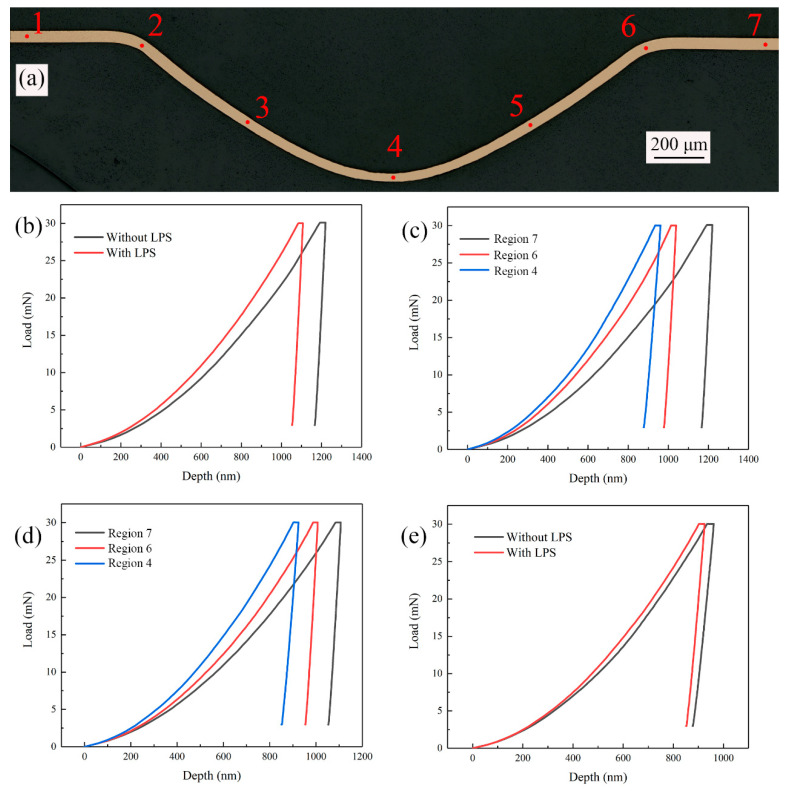
(**a**) Schematic diagram of measurement regions; (**b**) load-depth curves of unformed regions; (**c**) load-depth curves of different regions of the bulging sample without LPS; (**d**) load-depth curves of different regions of the bulging sample with LPS; (**e**) load-depth curves of bulging central regions.

In order to study the effect of LPS on microhardness and elastic modulus of bulging samples, the micro bulging experiment was carried out with laser pulse energy of 363 mJ and λ = 1. The measurement regions of the bulging sample are shown in Figure 16a. Region 1 and region 7 are unformed zone, region 2 and region 6 are the mold entrance zone, region 3 and region 5 are the bulging transition zone and region 4 is the bulging center zone. The Agilent Nano Indenter G200 nanoindentation in situ nanomechanics test system was used to test the bulging samples without LPS and with LPS. The indentation test points are measured every 0.2 s. The obtained load-depth curves of different regions are shown in Figure 16b–e. It can be found that the curves have no singularity and the experimental results are reliable. Compared with the measurement results of the unformed zone (shown in Figure 16b), the maximum indentation depth of bulging samples with LPS decreased from 1219.47 to 1106.81 nm. For bulging samples with LPS and without LPS, the variation trends of the maximum indentation depth at different regions in the bulging zone are similar. Generally, the closer to the bulging center zone, the smaller the maximum indentation depth (shown in Figure 16c,d). At the same time, the maximum indentation of the bulging sample with LPS is generally lower than that without LPS in the bulging zone. For example, after LPS, the maximum indentation depth in the bulging center zone decreases from 961.69 to 924.44 nm (as shown in Figure 16e).

**Figure 17:**
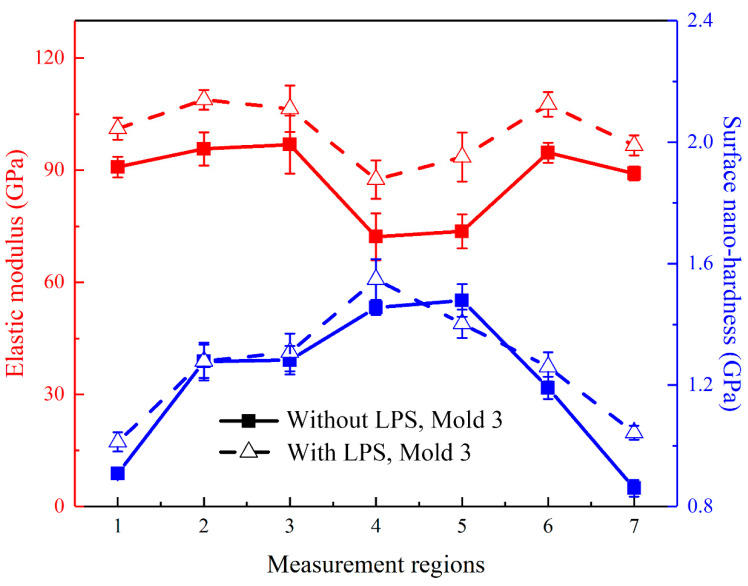
Surface nano-hardness and elastic modulus of different regions of bulging samples.

In the experiment, three points are selected for nanoindentation testing in each measurement region. The average nano-hardness of each measurement region calculated according to the obtained measurement curve is shown in Figure 17. For the bulging samples without LPS, the average nano-hardness in the measurement regions 1–7 is 0.908 GPa, 1.278 GPa, 1.282 GPa, 1.455 GPa, 1.479 GPa, 1.191 GPa and 0.860 GPa, respectively, and the average elastic modulus is 90.849 GPa, 95.723 GPa, 96.684 GPa, 72.194 GPa, 73.654 GPa, 94.679 GPa and 89.136 GPa, respectively. For the bulging samples with LPS, the average nano-hardness in each region is 1.013 GPa, 1.279 GPa 1.308 GPa, 1.548 GPa, 1.402 GPa, 1.260 GPa and 1.043 GPa, respectively, and the average elastic modulus is 101.115 GPa, 108.831 GPa, 106.448 GPa, 87.502 GPa, 93.502 GPa, 107.630 GPa and 96.643 GPa, respectively. It can be found that the nano-hardness of the bulging region (regions 2–6) is generally higher than that of the unformed region (regions 1 and 7), which is similar to the results obtained by Cheng et al. [39]. This is because with the increase of plastic strain, the movement of crystal dislocations becomes dense, resulting in the increase of hardness [40]. It is worth noting that the elastic modulus changes little in the unformed region and the mold entrance zone, but decreases significantly in the bulging zone. The reduced elastic modulus is related to the plastic strain [41]. After LPS, the grain of the workpiece is refined and the initial hardness is improved, thus the overall microhardness is high. By comparison, it is found that, for the bulging samples without LPS, the nano-hardness and elastic modulus change abnormally in the bulging transition zone (regions 3 and 5), which is consistent with the change trend of coincidence data in Figure 12a. At this time, the forming uniformity of bulging samples in the bulging transition zone is poor, which is due to the uneven microstructure in this zone [42]. LPS can generally improve the nano-hardness and elastic modulus of bulging samples [43], the change trend is more uniform and the forming uniformity can be improved.

## 4. Conclusions

In this paper, the effects of mold characteristic size and grain size on μLDFB were studied, and LPS is proposed as a pretreatment process. The effects of LPS were investigated from the aspects of surface roughness, forming depth, microstructure, forming symmetry, forming uniformity, nano-hardness and elastic modulus.

(1) For copper foil with constant thickness, there is a suitable mold characteristic size to obtain better forming quality (the mold diameter is 1.5 mm in this paper). The surface quality of bulging parts is poor when the mold characteristic size is small (the mold diameter is 1.0 mm). However, the forming symmetry and forming uniformity of bulging samples are reduced when the mold characteristic size is large (the mold diameter is 2.0 mm).
(2) The plastic strain increases with the increase of laser energy, and five stages can be observed: uniform plastic deformation, local necking, cracks in the bulging zone, complete fracture in the bulging zone and complete rupture at the mold entrance.
(3) The increase of grain size and mold characteristic size leads to the increase of surface roughening rate and local necking, which is the precursor of crack. The fracture mode of bulging samples belongs to ductile fracture.
(4) LPS plays a positive role in improving the surface quality and forming performance of bulging samples, which is mainly reflected in improving the forming symmetry and forming uniformity of bulging samples. The surface roughness of fine-grained bulging samples decreases from 2.267 to 1.797 μm with an increase of 20.7% (λ = 0.25, 363 mJ). Additionally, the maximum thinning rate of fine-grained bulging samples decreases from 67.5% to 24.5% (λ = 0.25, 575 mJ).
(5) The nano-hardness and elastic modulus of bulging samples without LPS change abnormally due to their uneven microstructure in the bulging transition zone, but there is no such phenomenon in bulging samples with LPS, which indicates that LPS can effectively improve the forming uniformity.

## Figures and Tables

**Table 1:** Grain size and N values of copper foils.

Values	450 °C Annealed, without LPS	450 °C Annealed, with LPS	650 °C Annealed, without LPS	650 °C Annealed, with LPS
Grain size (μm)	13.1	10.2	54.1	41.6
N values	3.8	4.9	0.9	1.2

**Table 2:** Design size of bulging molds.

Number	Diameter (mm)	Fillet (mm)	Scale Factor λ
1	1.0	0.10	0.25
2	1.5	0.15	0.5
3	2.0	0.20	1

**Table 3:** The technical parameters of the Spitlight 2000 Nd: YAG laser.

Parameters	Values
Laser pulse energy (mJ)	80–1800
Pulse width (ns)	8
Wave length (nm)	1064
Exit spot diameter (mm)	9

**Table 4:** Related experimental parameters.

Parameters	Values
Spot diameter (mm)	3
Mold diameter (mm)	1.0, 1.5, 2.0
PMMA thickness (mm)	3
Black paint thickness (μm)	~50
Soft film thickness (μm)	300
Laser pulse energy (mJ)	325, 363, 447, 575, 725, 1050, 1420

## Data Availability

Not applicable.
